# Obtention and Characterization of TiO_2_-Folic Acid-ZnPc Semiconductor Nanoparticles for Photodynamic Therapy Against Glioma Cells

**DOI:** 10.3390/pharmaceutics17081071

**Published:** 2025-08-19

**Authors:** Citlali Ekaterina Rodríguez-Pérez, Sonia Rodríguez-García, Ma. Elena Manríquez-Ramírez, A. Martin Ortiz-Torres, Francisco Tzompantzi-Morales, Emma Ortiz-Islas

**Affiliations:** 1 Laboratorio de Neurofarmacología Molecular y Nanotecnología, Instituto Nacional de Neurología y Neurocirugía, Insurgentes Sur 3877, La Fama, Tlalpan, Ciudad de México 14269, Mexico; crodriguez@innn.edu.mx (C.E.R.-P.); srrg24@comunidad.unam.mx (S.R.-G.); 2 Facultad de Psicología, Universidad Nacional Autónoma de México-UNAM, Av. Universidad 3004, Copilco, Ciudad Universitaria, Ciudad de México 04510, Mexico; 3 Laboratorio de Investigación en Nanomateriales y Energías Limpias, ESIQIE-IPN, Instituto Politécnico Nacional, Lindavista, Gustavo A. Madero, Ciudad de México 07700, Mexico; mmariquez@ipn.mx; 4 Instituto de Ingeniería de la UNAM, Circuito Escolar s/n, Ciudad Universitaria, Ciudad de México 04510, Mexico; aortizt@iingen.unam.mx; 5 Departamento de Química, Universidad Autónoma Metropolitana-Iztapalapa, San Rafael Atlixco 186, Vicentina, Iztapalapa, Ciudad de México 09340, Mexico; fjtz@xanum.uam.mx

**Keywords:** nanoparticles, TiO_2_, photosensitizers, semiconductors, photodynamic therapy, folic acid, zinc phthalocyanine, glioma cells

## Abstract

**Background/Objectives:** This study reports the synthesis of TiO_2_ nanoparticles, their functionalization with folic acid (FA), and the subsequent loading with zinc phthalocyanine (ZnPc) to develop photosensitizers for photodynamic therapy (PDT) targeting glioma cells. **Methods:** TiO_2_, TiO_2_-FA, and TiO_2_-FA-ZnPc nanoparticles were synthesized via a sol–gel process involving the hydrolysis and condensation of titanium (IV) isopropoxide. FA and ZnPc were incorporated in vitro during the synthesis. The resulting materials were characterized by transmission and scanning electron microscopy (TEM and SEM), X-ray diffraction (XRD), Raman and UV–Vis spectroscopy, thermogravimetric analysis (TGA), and nitrogen adsorption–desorption measurements. Reactive oxygen species (ROS) generation was evaluated in vitro using the 1,3-diphenylisobenzofuran (DPBF) probe. A 40 ppm solution of each TiO_2_ system was irradiated with UV light, and the degradation of DPBF was monitored. Biological assays were conducted to assess the viability of human glioblastoma cells (LN18 and U251) incubated with the TiO_2_-based materials, with and without UV exposure. Human fibroblast cells (BJ) were used to evaluate biocompatibility. **Results:** All TiO_2_-based materials retained key characteristics, including high surface area (~600–700 m^2^/g), mesoporous structure (pore diameter ~4–5 nm), mixed anatase–amorphous morphology, and a bandgap of approximately 3.46 eV. The UV–Vis spectrum of TiO_2_-FA-ZnPc displayed additional absorption bands in the visible region (600–700 nm), consistent with ZnPc incorporation. Upon UV irradiation, the DPBF absorbance at 410 nm decreased over time, indicating ROS generation and resulting in complete degradation within 10 min (TiO_2_), 12 min (TiO_2_-FA), and 14 min (TiO_2_-FA-ZnPc). BJ cells exhibited good biocompatibility at all concentrations. LN18 and U251 cells showed no cytotoxicity below 100 μg/mL unless exposed to UV light. **Conclusions:** The synthesized TiO_2_-based systems demonstrate good biocompatibility and significant phototoxicity under UV irradiation, highlighting their strong potential for application in photodynamic therapy.

## 1. Introduction

Titanium dioxide (TiO_2_), commonly known as titania, is a metal oxide widely used in photo-based applications due to its semiconducting properties. TiO_2_ naturally exists in three crystalline phases—anatase, rutile, and brookite—each exhibiting distinct physicochemical characteristics, structures, and morphologies [1]. Beyond its established use as a photocatalyst for organic pollutant degradation in wastewater, TiO_2_ is employed in hydrogen production via water splitting and in redox reactions to synthesize high-value products [2,3,4].

As a semiconductor, TiO_2_ becomes photoexcited upon UV or sunlight exposure. It absorbs photons with sufficient energy (≤390 nm), promoting electrons from the valence band to the conduction band, creating holes (h^+^) and electrons (e^−^). These charges can initiate photocatalytic oxidation-reduction reactions involving adsorbed molecules on the TiO_2_ surface [5]. In these reactions, water and molecular oxygen can interact with the surface charges to produce reactive oxygen species (ROS) such as hydroxyl radicals (•OH), superoxide anions (O_2_^−^), and hydrogen peroxide (H_2_O_2_). These ROS can degrade pollutants like dyes, pharmaceuticals, and pesticides, ultimately converting them into harmless byproducts like carbon dioxide and water [6,7,8]. This mechanism underpins the widespread use of TiO_2_ in environmental remediation technologies [9].

TiO_2_ also offers a large surface area, mesoporous structure, high stability, low toxicity, and good biocompatibility, making it attractive for biomedical applications [10,11,12]. It has been explored as a biomaterial in implants, biosensors, drug delivery systems, antimicrobial agents, and tissue engineering. Notably, TiO_2_’s ROS-generating capability under light irradiation has led to its investigation as a therapeutic agent in photodynamic therapy (PDT) for cancer treatment [13,14,15]. Upon activation by UV light, TiO_2_ can induce damage to cellular components such as DNA, membranes, and lipids, ultimately triggering cell death through apoptosis, necrosis, or autophagy. PDT applications of TiO_2_ have been reported effective against a variety of cancer cell lines, including breast (4T1), epithelial (KB), glioblastoma, non-small cell lung (A549), cervical (HeLa), and breast epithelial cells (MCF7, MDA-MB-231, NSCLC) [16,17,18,19,20]. Compared to traditional treatments, PDT is less invasive, with reduced toxicity and shorter treatment times, and is clinically approved. PDT may also complement surgery, chemotherapy, or radiotherapy.

**Scheme 1:**
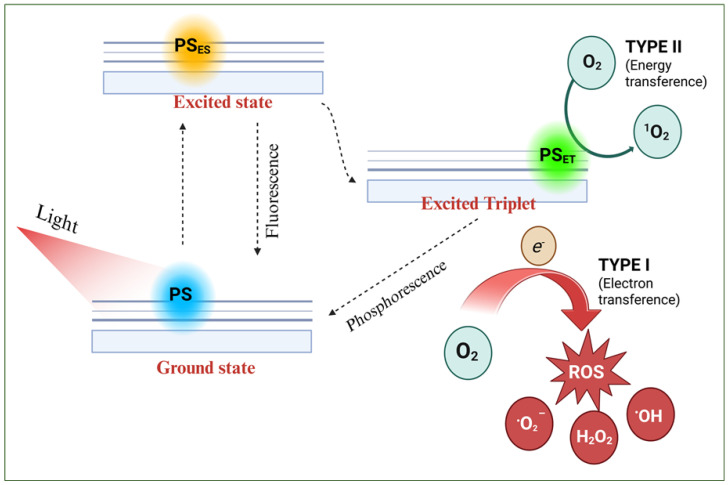
Schematic photodynamic reaction mechanisms (type I and II) upon light irradiation on a photosensitizer.

PDT relies on three key components: a light source (UV and/or visible), a photosensitizer (PS) that absorbs at the corresponding wavelengths, and sufficient molecular oxygen [21,22]. In practice, the PS is administered (orally, subcutaneously, or intravenously) and accumulates in the tumor. Once localized, it is irradiated to induce excitation from the ground state to higher energy states [21,22]. According to the literature, several processes can occur during excitation (see Scheme 1) [23]: (1) the PS transitions from its ground state to an excited singlet state (PSES); (2) this state may relax back to the ground state via fluorescence or non-radiative decay; or (3) it may convert to a longer-lived triplet state (PSET). The PSET species can then generate reactive oxygen species (ROS) via two pathways (Scheme 1): in a type I reaction, electron or proton transfer produces radicals that react with oxygen to form peroxides, hydroxyl radicals, or superoxide ions. In a type II reaction, energy is directly transferred to molecular oxygen, generating singlet oxygen. These ROS are cytotoxic and can induce apoptosis or necrosis in targeted cells [24].

Currently, a wide range of compounds are used as photosensitizers (PSs), including dyes, drugs, synthetic chemicals, and natural products. These are typically classified into first-, second-, and third-generation categories [25,26]. Early PDT applications used hematoporphyrin (HpD), a first-generation PS, which showed limitations such as short half-life and poor penetration into deep tissues [21]. This led to the development of second-generation PSs with improved properties and reduced toxicity. Most are heterocyclic porphyrins, including bacteriochlorins, chlorins, protoporphyrins, phthalocyanines, and their derivatives [21]. Non-porphyrin PSs include 5-aminolevulinic acid (ALA), curcumin, quinone, phenothiazine, and psoralen. Despite reduced toxicity, many second-generation PSs suffer from poor water solubility and low tumor selectivity. Third-generation PSs address these issues by modifying second-generation structures with bio-specific molecules or carriers to enhance selectivity and biocompatibility [27].

To date, only a few PSs have been approved for clinical use, most of which are based on the tetrapyrrole structure. Commercial examples include Photofrin, Levulan, Metvix, Cysview, Temoporfin, Verteporfin, Talaporfin, Photochlor, and Photosens, each with specific applications and limitations [28,29]. Photofrin, used clinically for over two decades, is approved for treating lung, gastric, bladder, cervical, and esophageal cancers. However, it has complex components, poor absorption in the therapeutic window, and a long clearance time. ALA (Levulan) is applied in cancer diagnosis and brain cancer research. Its low molecular weight and strong polarity make it suitable for topical use in dermatological conditions such as basal cell carcinoma, actinic keratosis, and Bowen’s disease. Metvix (MAL) and Cysview (HAL), esters of ALA, offer faster tissue penetration. Temoporfin is used against head and neck squamous cell carcinoma and shows improved light absorption and photodynamic effects but still requires 4–6 weeks of light avoidance post-treatment. Verteporfin, approved for age-related macular degeneration (AMD), marked a shift in PDT from oncology to ophthalmology. Talaporfin is primarily used in early-stage lung cancer and offers faster tumor accumulation and clearance than Photofrin. Photochlor has higher tumor uptake than Photofrin or Temoporfin due to its lipophilicity and better pharmacokinetics, with only mild, short-lived photosensitivity. Photosens, an aluminum phthalocyanine mixture, is approved in Russia for skin, stomach, and breast cancers and AMD. Hemoporfin is the first PS approved for port-wine stain (PWS) treatment, a congenital vascular malformation.

Nanomaterials as PS carriers—or as PSs themselves—present a promising strategy due to their generally low toxicity and high biocompatibility [30,31]. Their large surface area enables high PS loading and controlled release, enhances solubility, reduces aggregation, and improves ROS generation and light absorption for deeper tissue penetration. Surface functionalization with targeting ligands enables selective delivery to tumor cells, sparing healthy tissues [31,32]. Many cancer cells overexpress specific surface receptors, which can be targeted using ligands such as folate, CD44, monoclonal antibodies, and transferrin [33]. Nanomaterials for PDT are classified into two types: organic (e.g., liposomes, dendrimers, polymeric nanoparticles) and inorganic (e.g., silica, magnetic materials, quantum dots, metal oxides, and metals) [32].

According to the World Health Organization (WHO), glioblastoma is classified as a grade IV tumor, the most malignant form of brain cancer [34]. Although rare, it has a median survival of only 15 months, attributed to its high histological heterogeneity, invasiveness, rapid proliferation, and angiogenesis. Current standard treatment consists of maximal surgical resection followed by temozolomide (TMZ) chemotherapy and radiotherapy [35]. Despite these interventions, glioma patients face poor prognoses and limited survival, primarily due to the blood–brain barrier (BBB), which restricts the delivery of most chemotherapeutic agents. Consequently, there is a critical need to develop new or improved therapeutic strategies.

Phthalocyanines (Pcs), second-generation PSs, are aromatic heterocycles comprising four isoindole rings connected by nitrogen atoms. Their photophysical properties can be improved by introducing ring substituents or incorporating a silicon atom or metals such as zinc or aluminum [36]. Zinc phthalocyanine (ZnPc), which contains a zinc ion at its core, exhibits desirable characteristics including high ^1^O_2_ generation, strong therapeutic efficacy, low skin photosensitivity, and efficient ROS production. However, ZnPc has not yet been clinically approved due to its poor water solubility and aggregation tendency in solutions.

In this work, TiO_2_ semiconductor nanoparticles functionalized with folate groups and loaded with ZnPc were obtained, characterized, and applied as selective photosensitizers for glioma cells. The aim was to stabilize ZnPc on a functionalized TiO_2_-FA support that enables ROS generation and targeted delivery. The composite produced combines the beneficial properties of both TiO_2_ and ZnPc. Samples were synthesized using the sol–gel method, allowing in vitro incorporation of folic acid and ZnPc. Singlet oxygen generation was evaluated using the 1,3-diphenylisobenzofuran (DPBF) test, showing a decrease in the 410 nm band with increased UV irradiation and exposure to different TiO_2_ systems. LN18 and U251 cells showed no damage at low concentrations of these materials, compared to cells irradiated with UV light alone.

## 2. Materials and Methods

### 2.1. Sample Preparation

Chemical substances: tert-butyl alcohol [Sigma-Aldrich, St. Louis, MO, USA, ≥99%], titanium (IV) isopropoxide [Sigma-Aldrich, St. Louis, MO, USA, 97%], folic acid (FA) [Sigma-Aldrich, St. Louis, MO, USA, ≥97%], zinc phthalocyanine (ZnPc) [Aldrich, St. Louis, MO, USA, 97%], deionized water [Meyer, México City, México], and 1,3-diphenylisobenzofuran (DPBF) [Aldrich, St. Louis, MO, USA, 97%] were used as reagents.

**Scheme 2:**
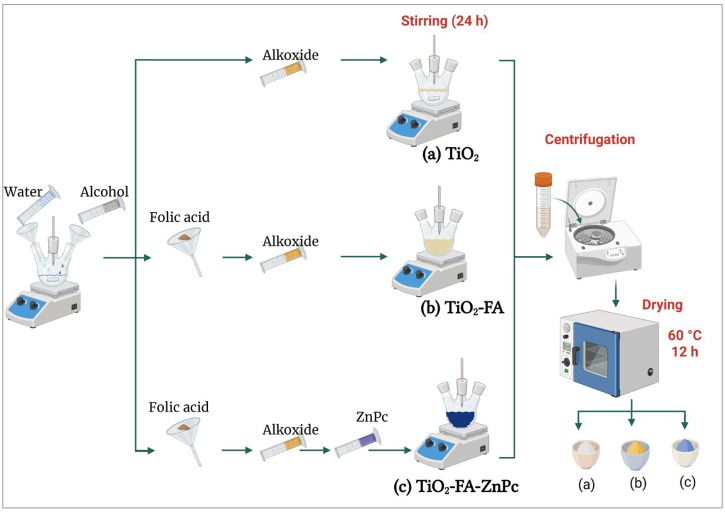
Graphical representation of the synthesis procedure of the different TiO_2_ powder samples: (**a**) TiO_2_, a white powder; (**b**) TiO_2_-FA, a yellow powder; and (**c**) TiO_2_-FA-ZnPc, a blue powder. These samples were prepared using the sol–gel method.

Three samples were synthesized via the sol–gel method: pure TiO_2_, TiO_2_ functionalized with folic acid (TiO_2_-FA), and TiO_2_-FA loaded with zinc phthalocyanine (TiO_2_-FA-ZnPc). The molar ratios were alkoxide:water (1:16) and alkoxide:alcohol (1:8). A total of 0.5 g of FA and ZnPc was added. The synthesis procedure is illustrated in Scheme 2.

#### 2.1.1. TiO_2_

A solution of 185 mL tert-butyl alcohol and 72 mL deionized water was stirred for 30 min. Then, 78 mL of titanium (IV) isopropoxide was added dropwise over 4 h. After complete addition, the mixture was stirred for 24 h. Excess water and alcohol were removed by centrifugation, and the resulting solid was dried at 60 °C for 12 h.

#### 2.1.2. TiO_2_-FA

Folic acid (0.5 g) was dissolved in 185 mL tert-butyl alcohol and 72 mL water. Then, 78 mL of titanium (IV) isopropoxide was added dropwise. The mixture was stirred for 24 h, centrifuged to remove solvents, and dried at 60 °C for 12 h.

#### 2.1.3. TiO_2_-AF-ZnPc

Folic acid (0.5 g) was dissolved in 92.6 mL tert-butyl alcohol and 36 mL water and stirred for 30 min. Then, 78 mL of titanium (IV) isopropoxide was added dropwise and the mixture stirred for 5 h. A solution containing 0.5 g zinc phthalocyanine in 92.6 mL tert-butyl alcohol and 36 mL water was then added. The mixture was stirred for 24 h and dried at 60 °C for 12 h.

### 2.2. Samples Characterization

Scanning electron microscopy (SEM) was conducted using a Schottky JSM-7800F field-emission microscope (Tokyo, Japan) to observe morphology and surface texture. Images were acquired with a secondary electron detector at 2.0 kV under ultra-high vacuum. Particle dimensions were measured using ImageJ software (https://share.google/M9IyYsVqNLaGpH9Xa, accessed on 9 and 26 September 2024). High-resolution transmission electron microscopy (HR-TEM) was carried out on a JEM-2100 microscope (Tokyo, Japan) (LaB_6_ filament, 80–200 kV). Samples were dispersed in isopropanol by sonication and deposited onto copper grids for analysis. X-ray diffraction (XRD) patterns were obtained using a Bruker D2 Phaser diffractometer (Karlsruhe, Germany,) with Cu Kα radiation (λ = 1.5405 nm). Scans were conducted from 7° to 80° (2θ) at a rate of 0.0405°/s. Samples were pressed into the holder to ensure a flat, uniform surface. Raman spectra were recorded at room temperature using a Jobin Yvon HR800 micro-Raman spectrometer (Palaiseau, France) with a 532 nm excitation laser and D3 filter. Spectra were collected in the range of 0–3500 cm^−1^ with a 2 s exposure time. Powdered samples were placed in clean glass holders. Diffuse reflectance UV–Vis spectra were recorded using a Varian Cary 100 Scan spectrophotometer (Palo Alto, CA, USA) equipped with a calcium sulfate integrating sphere, covering the 190–800 nm range. Thermogravimetric analysis (TGA) was performed using an SAT-i 1000 instrument (Twin Lakes, WI, USA). Samples were heated from room temperature to 800 °C at a rate of 10 °C/min under nitrogen to assess thermal stability and weight loss. Nitrogen adsorption–desorption isotherms were measured using a Belsorp-mini II system (Osaka, Japan). Samples were degassed at 60 °C under vacuum for 12 h. Surface area, pore volume, and pore size were calculated from the resulting isotherms.

### 2.3. In Vitro ROS Determination

1,3-Diphenylisobenzofuran (DPBF) was used as a fluorescent probe to evaluate singlet oxygen formation. Briefly, 200 mL of a 40 ppm DPBF solution was placed in a custom-made photoreactor equipped with water recirculation and air flow. The solution was bubbled with air for 20 min. Subsequently, 200 mg of the TiO_2_ system was added and allowed to interact with the solution for 5 min. The mixture was then irradiated with a UV lamp at 254 nm (2.2 mW, UVP company, Upland, CA, USA). Aliquots were collected every 2 min for analysis by UV–Vis spectroscopy using a Cary100SCAN UV–Vis spectrophotometer (Palo Alto, CA, USA).

### 2.4. Biological Assays

Cell culture substances: Tetrazolium bromide (MTT) (Sigma-Aldrich, ≥97%), fetal bovine serum (FBS) (Biowest, Nuaillé, France), Dulbecco’s Modified Eagle Medium (DMEM) (Gibco, Waltham, MA, USA), trypsin-EDTA (Gibco), and antibiotic-antimycotic (Corning, Somerville, MA, USA).

Cell line: LN18 and U251 (human glioblastoma) and BJ (human fibroblasts) obtained from ATCC.

#### 2.4.1. Cytotoxic Evaluation

To assess the cytotoxicity of the developed TiO_2_ systems, cell viability was measured using the MTT (3-(4,5-dimethylthiazol-2-yl)-2,5-diphenyltetrazolium bromide) assay on human fibroblast BJ cells and glioblastoma cell lines LN18 and U251.

Cells were seeded at a density of 1 × 10^5^ cells/well in 96-well plates and incubated overnight at 37 °C in a humidified 5% CO_2_ atmosphere. The following day, cells were treated with increasing concentrations (0, 10, 25, 50, 100, and 500 μg/mL) of TiO_2_, TiO_2_-FA, and TiO_2_-FA-ZnPc in DMEM supplemented with 5% FBS. Both treated and untreated cells were incubated for 24 h. MTT reagent was added and incubated for 2 h to allow formazan crystal formation in metabolically active cells. Subsequently, 100 μL of acidified isopropanol (0.04 M HCl) was added to dissolve the crystals. Absorbance was measured at 570 and 630 nm using a microplate reader.

Cell viability was calculated using the following formula:

*CV*% = (*S*(*A*570 − *A*630))/(*C*(*A*570 − *A*630)) × 100%

where

*CV* is the cell viability, *S* is the sample, *C* represents the control cells, and *A* is the absorbance.

Experiments were performed in triplicate, and controls consisted of untreated cells. Results are expressed as percentages relative to the control group. Statistical analysis was conducted using two-way ANOVA with Dunnett’s post hoc test (GraphPad Prism, version 9.4.1).

#### 2.4.2. Determination of the Phototoxicity Effect

To assess phototoxicity and distinguish treatment toxicity from light-induced cytotoxicity, LN18 and U251 glioma cells were seeded at a density of 1 × 10^5^ cells/well in 96-well plates and incubated overnight at 37 °C in a humidified atmosphere with 5% CO_2_ to allow cell adhesion. The following day, cells were treated with increasing concentrations (0, 10, 25, 50, 100, 250, and 500 μg/mL) of each nanoparticle formulation and dissolved in DMEM supplemented with FBS. After 10 min of incubation, cells were irradiated with 385 nm LED light for either 1 or 10 min at distances of 1 and 10 cm. Irradiation was performed inside a laminar flow hood to prevent contamination and ensure consistent exposure conditions.

Following irradiation, cells were incubated for 24 h at 37 °C with 5% CO_2_. Cell viability was determined using the MTT colorimetric assay. MTT was added to each well and incubated for 2 h to allow formazan crystal formation. Crystals were then solubilized with acidified isopropanol (0.04 M HCl), and absorbance was measured at 570 and 630 nm using a microplate reader. All treatments were conducted in triplicate. Results were expressed as percentage viability relative to untreated controls. Statistical analysis was performed using two-way ANOVA with Dunnett’s post hoc test and paired Student’s *t*-test for specific comparisons (GraphPad Prism, version 9.4.1).

## 3. Results

### 3.1. Characterization Results

**Figure 1:**
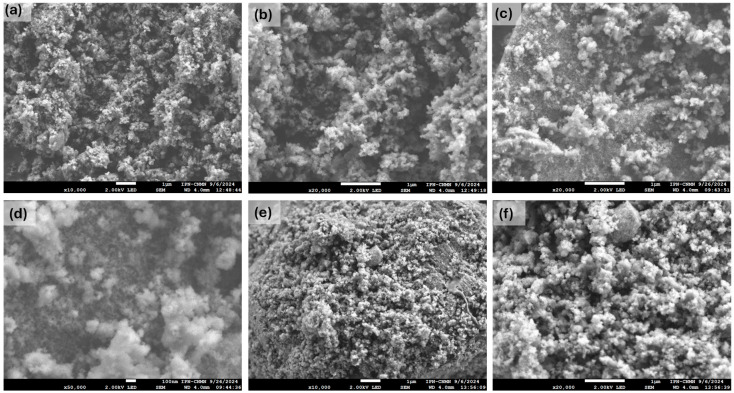
SEM images at different magnifications of the different prepared TiO_2_ materials: (**a**) TiO_2_ (×10,000), (**b**) TiO_2_ (×20,000), (**c**) TiO_2_-FA (×20,000), (**d**) TiO_2_-FA (×50,000), (**e**) TiO_2_-FA-ZnPc (×10,000), and (**f**) TiO_2_-FA-ZnPc (×20,000). The scale bar in the (**d**) sample corresponds to 100 nm; the rest correspond to 1 µm.

SEM images of the various TiO_2_ samples at different magnifications are shown in Figure 1. The TiO_2_ sample displays a morphology composed of agglomerates of small particles ranging from approximately 10 to 100 nm (Figure 1a,b), forming a porous TiO_2_ network. Similarly, the TiO_2_-FA sample exhibits a morphology of aggregated particles (Figure 1c,d), and the TiO_2_-FA-ZnPc sample shows the same structural characteristics (Figure 1e,f). In all cases, the nanoparticle aggregates result in a porous morphology, typical of TiO_2_ materials synthesized by the sol–gel method.

**Figure 2:**
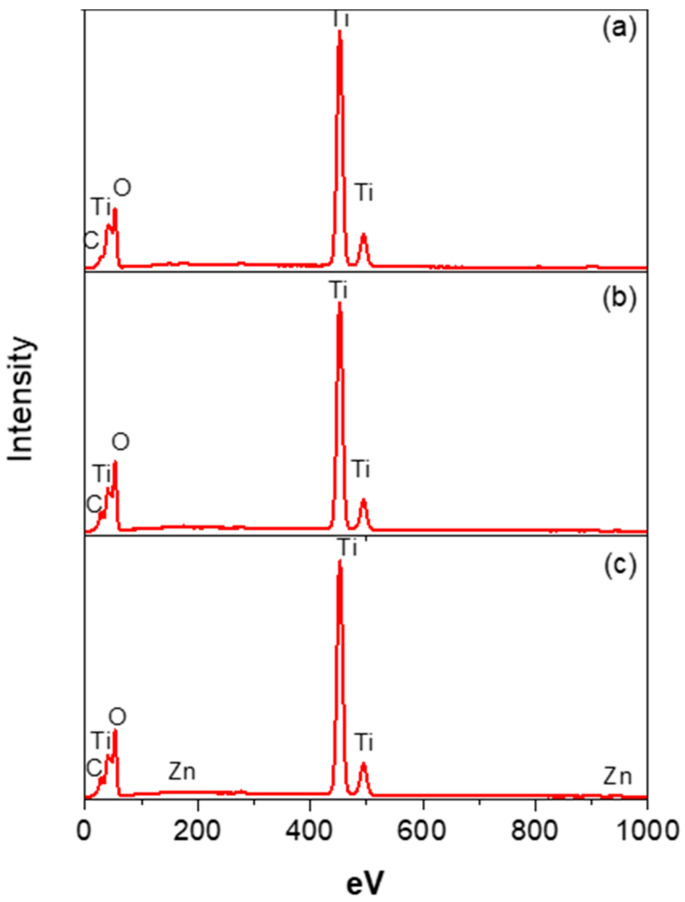
EDS spectra of the different TiO_2_-based samples, identifying the main Ti, O, C, and Zn elements present in (**a**) TiO_2_, (**b**) TiO_2_-FA, and (**c**) TiO_2_-ZnPc samples.

Figure 2 presents the energy-dispersive X-ray spectroscopy (EDS) spectra of the TiO_2_, TiO_2_-FA, and TiO_2_-FA-ZnPc samples. The EDS spectrum of the TiO_2_ sample indicates titanium as the primary element, along with smaller signals for oxygen and carbon. Titanium and oxygen are part of the TiO_2_ network, while the carbon likely originates from the alkoxide precursor used in the synthesis (Figure 2a). The TiO_2_-FA spectrum is similar, showing Ti, O, and a stronger C signal, the latter attributed both to the alkoxide precursor and to folic acid (Figure 2b). The TiO_2_-FA-ZnPc spectrum displays peaks for carbon, oxygen, and titanium, along with a small zinc signal (Figure 2c), indicating the presence of Zn from the ZnPc component. However, Ti, O, and C remain the dominant elements in all samples.

**Figure 3:**
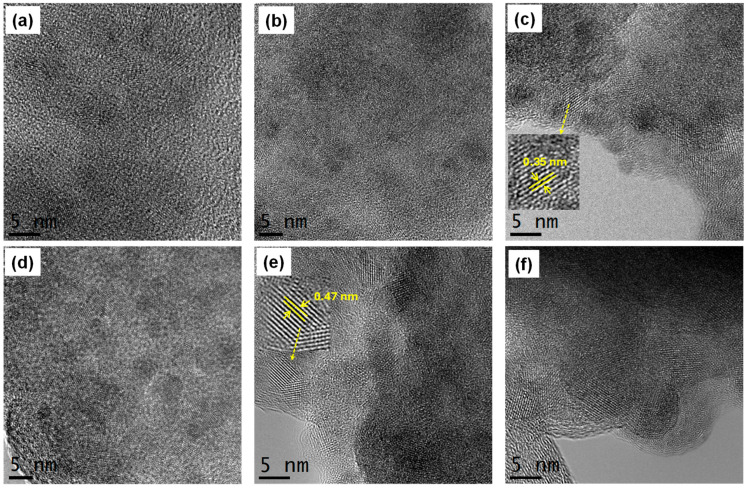
HR-TEM images of (**a**,**b**) TiO_2_, (**c**,**d**) TiO_2_-FA, and (**e**,**f**) TiO_2_-ZnPc. Zoomed regions show the interplanar spacing of (**c**) anatase phase and (**e**) ZnPc, respectively.

Figure 3 shows high-resolution transmission electron microscopy (HR-TEM) images of the different TiO_2_-based materials prepared as photosensitizers. The TiO_2_ sample exhibits a combination of amorphous and crystalline regions (Figure 3a,b), with the crystalline areas likely corresponding to the anatase phase. In the TiO_2_ sample functionalized with folic acid (TiO_2_-FA), an increased degree of crystallinity is observed, suggesting that folic acid promotes anatase phase formation (Figure 3b,c). Upon the addition of ZnPc, well-defined crystalline domains appear, although amorphous regions remain visible across all samples. The HR-TEM images were also used to measure the interplanar spacing of the TiO_2_-FA and TiO_2_-FA-ZnPc samples. For TiO_2_-FA, an interplanar spacing of approximately 0.35 nm was calculated, corresponding to the (101) plane of anatase (Figure 3c). In the TiO_2_-FA-ZnPc sample (Figure 3e), a distinct crystalline region displays a lattice spacing of 0.47 nm, which matches the (202) plane of ZnPc and is consistent with reported values [37].

**Figure 4:**
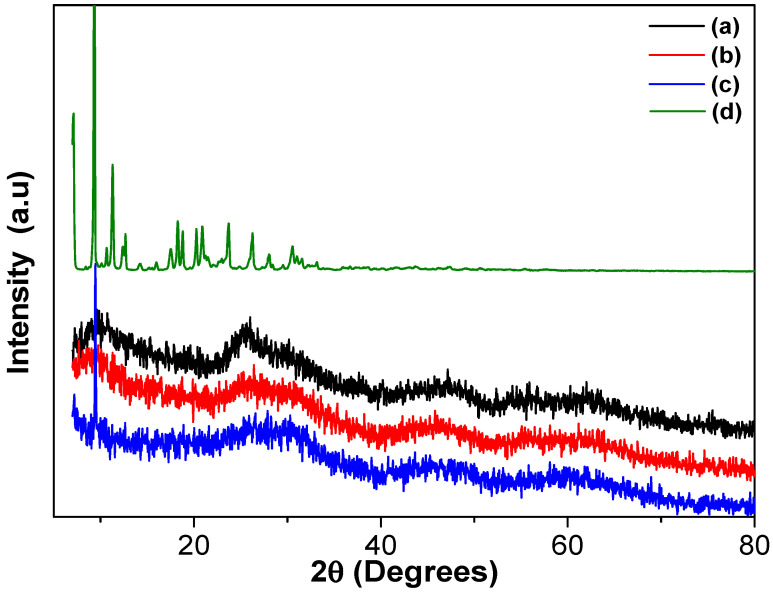
XRD patterns of the different TiO_2_ materials: (a) TiO_2_, (b) TiO_2_-FA, (c) TiO_2_-FA-ZnPc, and (d) ZnPc.

Figure 4 presents the X-ray diffraction (XRD) patterns of the studied materials. The TiO_2_ pattern reveals a combination of amorphous and anatase phases, evidenced by the peak at 25.2°, corresponding to the (101) plane of anatase. The ZnPc pattern displays sharp peaks indicative of a crystalline structure. The most intense peak appears at 2θ = 9.34°, accompanied by medium-intensity peaks at 2θ = 7.08° and 11.28°, and several minor peaks at 2θ = 12.66°, 18.28°, 18.78°, 20.26°, 20.91°, 23.74°, and 26.29°, which correspond to various ZnPc diffraction planes [37,38]. In the TiO_2_-FA-ZnPc diffractogram, the features of TiO_2_ predominate, but the peak at 2θ = 9.34°, associated with ZnPc, remains visible, indicating the successful incorporation of ZnPc into the TiO_2_ network. The TiO_2_-FA pattern closely resembles that of TiO_2_, suggesting that folic acid is well distributed within the porous titania matrix.

**Figure 5:**
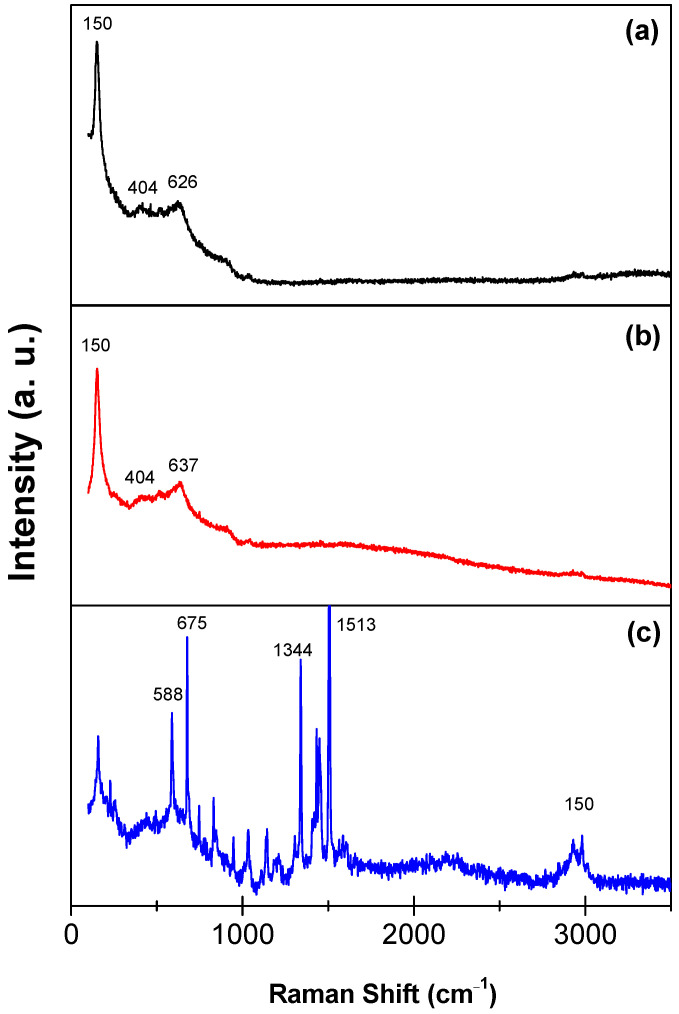
Raman spectra of the (**a**) TiO_2_, (**b**) TiO_2_-FA, and (**c**) TiO_2_-FA-ZnPc materials.

Figure 5 shows the Raman spectra of the TiO_2_, TiO_2_-FA, and TiO_2_-FA-ZnPc samples over the 0–3500 cm^−1^ range. In Figure 5a, the TiO_2_ sample displays a strong, well-defined peak at 150 cm^−1^, attributed to the Eg(2) mode of the anatase crystalline phase. The band at 623 cm^−1^ corresponds to another Eg mode, and the peak at 404 cm^−1^ is assigned to the B1g mode of anatase [39]. The Raman spectrum of TiO_2_-FA is very similar to that of TiO_2_, with a slight shift of the band at 637 cm^−1^ due to the presence of folic acid (Figure 5b), indicating its good dispersion within the TiO_2_ network. In the spectrum of TiO_2_-FA-ZnPc (Figure 5c), additional distinct peaks appear alongside the TiO_2_-related bands. According to the literature, the peak at 588 cm^−1^ arises from benzene ring deformation, the 675 cm^−1^ peak is attributed to the macrocycle, and the bands at 1344 cm^−1^ and 1513 cm^−1^ are associated with the Zn metal ion coordinated to the phthalocyanine structure [40,41]. In the 2000–3800 cm^−1^ region, carbon-related vibrational modes from ZnPc are also evident.

**Figure 6:**
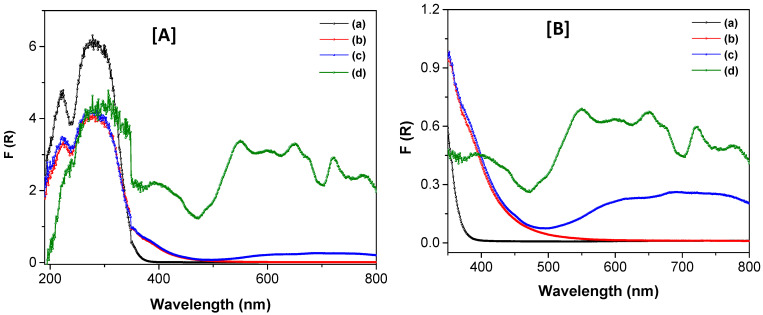
UV–Vis spectra of the different TiO_2_-based materials in their solid state (**A**) in the 200–800 range and (**B**) in the 350–800 range, respectively. (a) TiO_2_, (b), TiO_2_-FA, (c) TiO_2_-FA-ZnPc, and (d) ZnPc.

Figure 6 shows the UV–Vis diffuse reflectance spectra in the solid state for TiO_2_, TiO_2_-FA, and TiO_2_-FA-ZnPc materials. The TiO_2_ spectrum exhibits a strong absorption band at 380 nm (Figure 6A), corresponding to an electron transition from the Ti 3d orbital to the O 2p orbitals in the TiO_2_ lattice [42]. The spectrum of TiO_2_-FA closely resembles that of TiO_2_; however, the main absorption peak is red-shifted from 380 nm to 520 nm, likely due to interactions between folic acid and the TiO_2_ surface. The ZnPc spectrum displays multiple absorption bands throughout the UV–Vis range, which are categorized into the visible and ultraviolet regions. In the visible range, signals between 450–750 nm correspond to the Q-band (λ = 660 nm), while the ultraviolet range features the B-band (λ = 327 nm) [43]. The Q-band is attributed to p-p* transitions from the highest occupied molecular orbital (HOMO) to the lowest unoccupied molecular orbital (LUMO) in ZnPc. The B-band arises from transitions from deeper p orbitals to the LUMO [44]. In the TiO_2_-FA-ZnPc spectrum, the Q-band is visible in the magnified region of Figure 5b. The B-band is not discernible, possibly due to overlap with TiO_2_ absorption. The experimental bandgap energy (Eg) for TiO_2_ was calculated as 3.46 eV, whereas the Eg for both TiO_2_-FA and TiO_2_-FA-ZnPc was 2.38 eV. Notably, absorption in the visible region is evident in the TiO_2_-FA-ZnPc spectrum.

**Figure 7:**
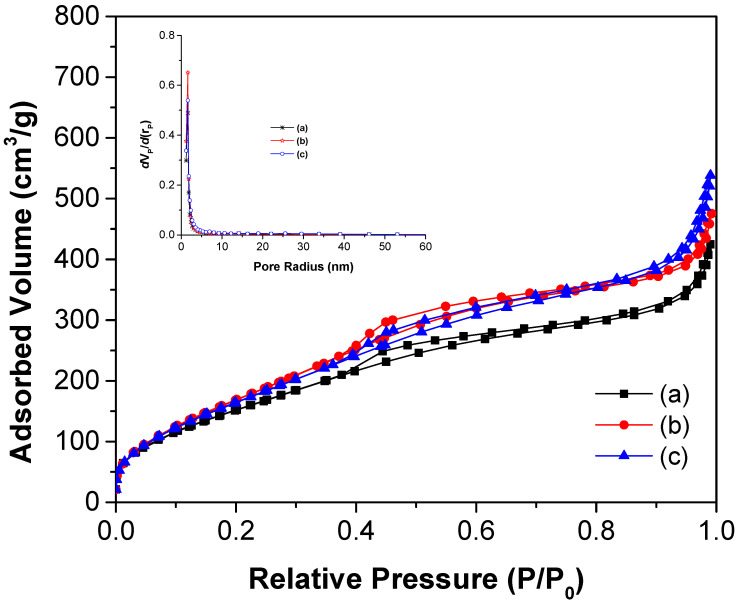
N_2_ adsorption–desorption isotherms of (a) TiO_2_, (b) TiO_2_-FA, and (c) TiO_2_-FA-ZnPc materials. The insert shows the pore size distribution of the materials.

Figure 7 presents the nitrogen adsorption–desorption isotherms for the synthesized TiO_2_ materials. Based on IUPAC classification, all curves correspond to Type IV isotherms, characteristic of mesoporous materials. A hysteresis loop of type H1, also defined by IUPAC, confirms the presence of mesoporosity. At low relative pressure (P/P_0_), the adsorbed volume between 0.01–0.2 nm indicates the presence of micropores, which are further supported by the pore size distribution shown in the inset of Figure 7. Additional uptake at high P/P_0_ values (0.95–1.0) suggests the presence of macropores, albeit in smaller amounts compared to micro- and mesopores. Overall, mesopores dominate the porosity profile of the materials.

Textural properties such as surface area, average pore diameter, and pore volume were derived from nitrogen adsorption–desorption isotherms and are summarized in Table 1. The surface area for TiO_2_ was 602 nm, while those for TiO_2_-FA and TiO_2_-FA-ZnPc were 706 and 675 nm, respectively. All values are relatively high. Notably, the surface area increased upon the addition of folic acid. However, when ZnPc was incorporated, the surface area decreased by 4.31% compared to the TiO_2_-FA sample. This reduction may be due to ZnPc molecules occupying part of the pores and surface, as they were added after the TiO_2_-FA network had formed. The average pore diameter decreased from 5.35 nm in TiO_2_ to 4.07 nm in TiO_2_-FA, likely due to folic acid molecules occupying the pore spaces. The pore volume followed the trend TiO_2_-FA-ZnPc > TiO_2_-FA > TiO_2_, with all materials exhibiting relatively high pore volumes.

**Figure 8:**
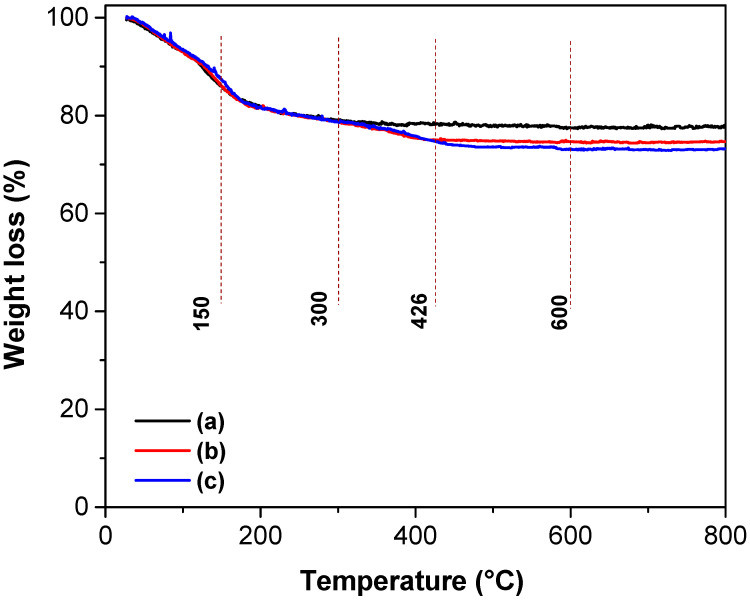
TGA curves of the different TiO_2_-based materials: (a) TiO_2_, (b) TiO_2_-FA, and (c) TiO_2_-FA-ZnPc.

The weight loss profiles as a function of treatment temperature for the TiO_2_, TiO_2_-FA, and TiO_2_-FA-ZnPc samples are shown in Figure 8, with the corresponding percentage weight loss values for each temperature range summarized in Table 2. The thermogram of TiO_2_ exhibits two major weight loss events. The first, occurring between room temperature and 150 °C, is attributed to the evaporation of volatile compounds such as water and alcohol used during synthesis, resulting in a 14% weight loss (Table 2). The second event, between 150–300 °C, likely corresponds to the decomposition of residual precursor materials such as alkoxides, accounting for an additional 8% loss. Beyond this temperature range, the thermogram becomes linear, indicating no further significant weight loss.

The thermogram of TiO_2_-FA reveals three distinct stages of weight loss. The first stage, from room temperature to 150 °C, corresponds to the evaporation of water and alcohol, also resulting in a 14% weight loss. The second stage, from 150–300 °C, involves an 8% loss attributed to the removal of organic precursors. The third stage, from 300–426 °C, accounts for a 3% weight loss due to the complete degradation of folic acid. Following this, the thermogram becomes stable and linear, indicating no additional mass loss. In total, 20 g of TiO_2_ were synthesized, and 0.5 g of folic acid was added, representing 2.5% of the TiO_2_ mass. The 3% weight loss attributed to folic acid (Table 2) closely matches the theoretical value, confirming the sample’s composition by TGA analysis.

The thermogram of the TiO_2_-FA-ZnPc sample displays four main stages of weight loss. As in the previous sample, the first three stages correspond to (1) the evaporation of water and alcohol and (2–3) the elimination of folic acid, showing weight loss percentages similar to those of TiO_2_-FA (Table 2). The fourth stage, occurring between 426–600 °C, corresponds to the degradation of ZnPc and results in a 2% weight loss. This value is consistent with the 2.5% ZnPc added during synthesis, further validating the composition of the sample.

### 3.2. In Vitro ROS Production

**Figure 9:**
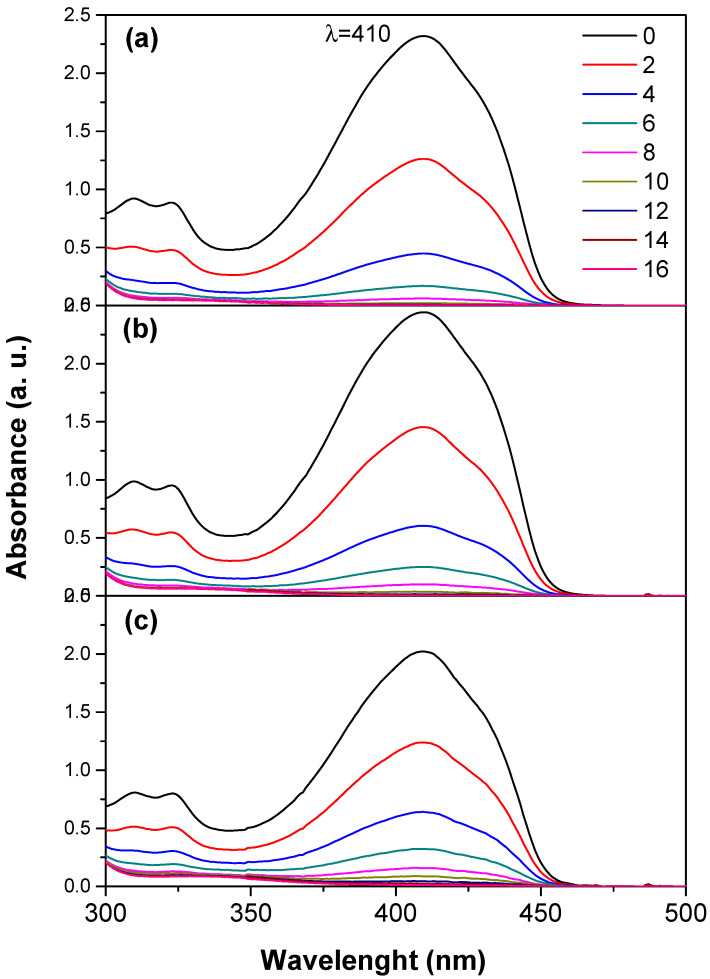
UV–Vis spectra of the 1,3-diphenylisobenzofuran (DPBF) in the presence of the TiO_2_ samples after irradiation with UV light for different times (0–16 min). (**a**) TiO_2_, (**b**) TiO_2_-FA, (**c**) TiO_2_-FA-ZnPc.

The generation of singlet oxygen was confirmed using 1,3-diphenylisobenzofuran (DPBF) as a probe. Figure 9 shows the UV–Vis absorption spectra of DPBF in the presence of TiO_2_, TiO_2_-FA, and TiO_2_-FA-ZnPc after UV light irradiation for up to 16 min. The absorption intensity of DPBF gradually decreased over time, accompanied by a visible color change from green to colorless. Complete degradation of DPBF was observed after 10 min of irradiation with TiO_2_, 12 min with TiO_2_-FA, and 14 min with TiO_2_-FA-ZnPc.

### 3.3. Biological Results

**Figure 10:**
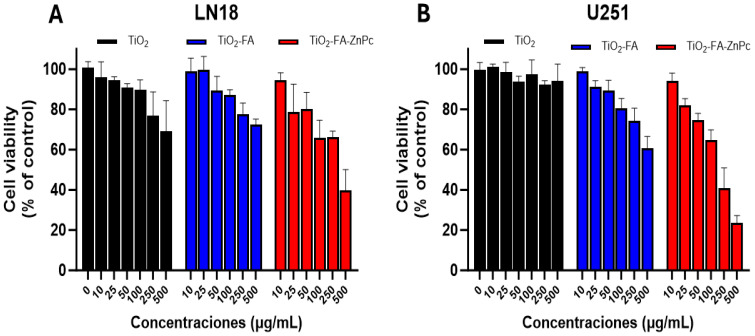
Viability of (**A**) LN18 and (**B**) U251 cells after treatment with different concentrations of TiO_2_ (black), TiO_2_-FA (blue), and TiO_2_-FA-ZnPc (red) nanoparticles (24 h). The standard deviations are indicated with the error bars.

**Figure 11:**
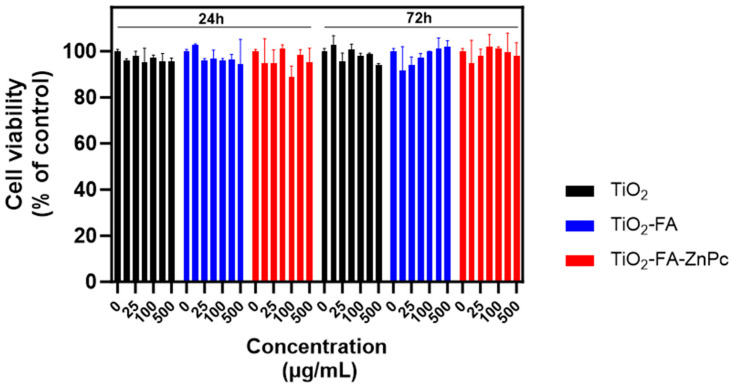
Viability of BJ cells after treatment with different concentrations of TiO_2_, TiO_2_-FA, and TiO_2_-FA-ZnPc nanoparticles (24 h and 72 h). The standard deviations are indicated with the error bars.

The viability of LN18 and U251 cells was evaluated using the MTT assay after 24 h of exposure to TiO_2_, TiO_2_-FA, and TiO_2_-FA-ZnPc materials (Figure 10), as well as in BJ fibroblasts (Figure 11). This assay quantifies cell metabolic activity and serves as an indicator of cytotoxicity. The results show that the average viability of LN18 cells exposed to varying concentrations of the TiO_2_-based materials (5–100 µg/mL) decreased with increasing TiO_2_ concentration, following a dose-dependent trend over time. After 24 h, cell viability remained high at low and moderate concentrations (≤100 µg/mL) for all three materials, indicating good initial biocompatibility (Figure 10A,B). However, at concentrations of 250 µg/mL and above, significant cytotoxic effects were observed.

At the lowest concentrations tested (10–100 µg/mL), cell viability remained relatively high, showing no significant differences from the control group. In contrast, at concentrations between 250 and 500 µg/mL, a statistically significant reduction in cell viability was detected, particularly in the TiO_2_-FA-ZnPc group. Notable differences began to emerge at 250 µg/mL (*p* < 0.05) according to Dunnett’s analysis.

Figure 11 shows the cell viability of BJ fibroblasts (used as normal cells) treated with increasing concentrations of the different TiO_2_ samples for 24 and 72 h. As observed in each group, viability remained comparable to the control (cells without treatment) across all tested concentrations and exposure times. These results confirm that the synthesized materials are biocompatible.

**Figure 12:**
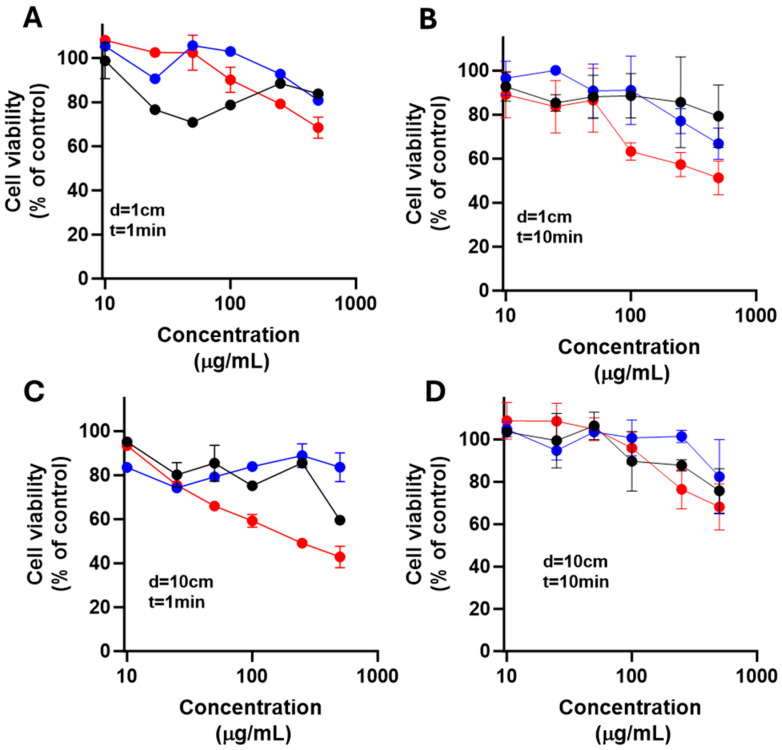
Effect of nanoparticles over cell viability after irradiation on LN18 cells. The cells were exposed to LED light at 385 nm for 1 min (**A**,**C**) or 10 min (**B**,**D**) with 1 cm (**A**,**B**) and 10 cm (**C**,**D**) from irradiation source after placing TiO_2_ (black), TiO_2_-FA (blue), and TiO_2_-FA-ZnPc (red) nanoparticles in cells; followed; after 24 h, cell viability was determined. Results are expressed as means ± SEM.

**Figure 13:**
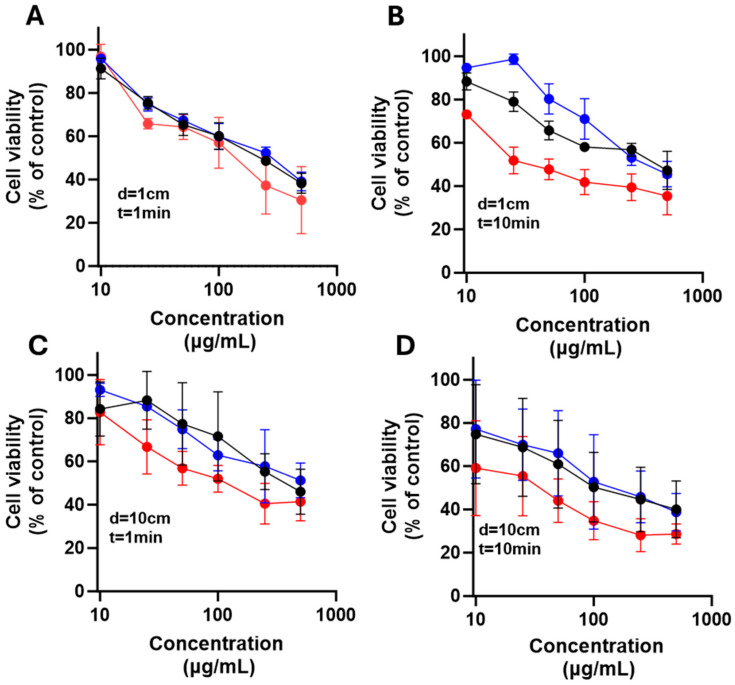
Effect of nanoparticles over cell viability after irradiation on U251 cells. The cells were exposed to LED light at 385 nm for 1 min (**A**,**C**) or 10 min (**B**,**D**) with 1 cm (**A**,**B**) and 10 cm (**C**,**D**) from the irradiation source after placing TiO_2_ (black), TiO_2_-FA (blue), and TiO_2_-FA-ZnPc (red) nanoparticles in cells; followed; after 24 h, cell viability was determined. Results are expressed as means ± SEM.

As shown in Figure 12 and Figure 13, TiO_2_-ZnPc nanoparticles combined with UV irradiation enhanced cell death from 100 µg/mL under all conditions, although LN18 cells showed greater resistance compared to U251 cells. The U251 cells exhibited a dose- and light-dependent differential response at 10 cm and with increased irradiation time.

Cells were exposed to LED light at 385 nm for 1 min (A and C) or 10 min (B and D), using distances of 1 cm (A and B) and 10 cm (C and D) from the irradiation source. After 24 h, cell viability was determined. Results are expressed as means ± SEM.

## 4. Discussion

**Scheme 3:**
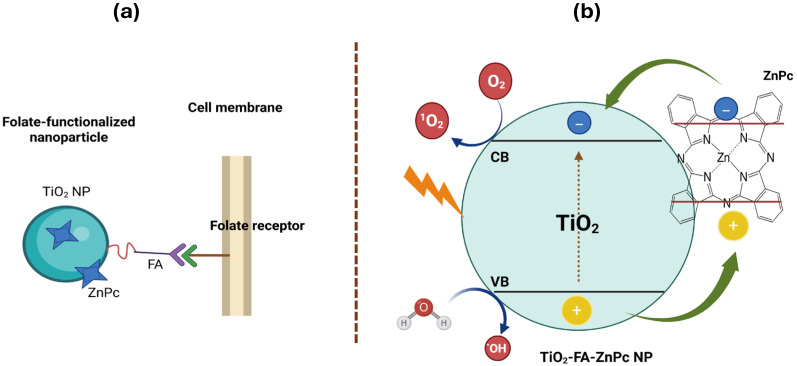
(**a**) A schematic representation of the functionalization of a TiO_2_ nanoparticle with folic acid and loaded with ZnPc and its subsequent entry into a cell via folate receptors and (**b**) the ROS formation when TiO_2_-FA-ZnPc nanoparticle is irradiated with UV light.

Folic acid, also known as vitamin B9, is a water-soluble vitamin essential for the synthesis of nitrogenous bases during nucleotide formation [45]. Cancer cells require increased amounts of folate for proliferation and survival, often overexpressing folate receptors to enhance uptake. Therefore, folic acid is commonly used to functionalize nanomaterial surfaces for selective cancer cell targeting [46]. Folate-functionalized nanomaterials enter cancer cells via folate receptor-mediated endocytosis (Scheme 3a). This approach is feasible because most healthy tissues exhibit low folate receptor expression, allowing discrimination between cancerous and normal cells. Accordingly, the TiO_2_-FA system was designed to enable selective interaction with glioma cells through folate receptor binding.

Initially, TiO_2_ nanoparticles were synthesized, exhibiting desirable physicochemical properties including nanostructured morphology, high surface area (602 m^2^/g), mesoporous structure (average pore size 5.35 nm), a mixed anatase-amorphous phase, and a bandgap of 3.46 eV—typical for semiconductor TiO_2_. These properties facilitated the uniform incorporation and dispersion of folic acid throughout the TiO_2_ network, resulting in a composite with distinct features: a larger surface area of 706 m^2^/g, preserved mesoporosity (4.07 nm), and the same mixed-phase composition. This was achieved via the sol–gel method, widely used for homogeneous in situ incorporation of active species, eliminating post-synthesis modifications [47]. Thermogravimetric analysis confirmed that the incorporated amounts of folic acid and ZnPc closely matched their theoretical values (Table 2). A photosensitizing nanocomposite was subsequently obtained by loading ZnPc onto the TiO_2_-FA structure. ZnPc can remain both on the surface and/or embedded within the titania lattice. The final TiO_2_-FA-ZnPc composite retained its unique properties while enhancing the functionalities of both TiO_2_ and ZnPc. This material had a high surface area (675 m^2^/g) and strong absorption in both UV and visible regions, resulting in a reduced bandgap of 2.3 eV.

Titania is a well-known semiconductor that generates significant amounts of reactive oxygen species (ROS) under UV irradiation (Scheme 3). However, when TiO_2_ is combined with dye molecules, its bandgap changes substantially due to photosensitization [48]. This lowers the energy required to excite electrons, allowing light absorption at higher wavelengths. As a result, TiO_2_-FA-ZnPc can be activated not only by UV light but also by visible radiation. Therefore, sunlight and other visible light sources can photoexcite TiO_2_-FA-ZnPc materials. As illustrated in Scheme 3b, when the photosensitizer (TiO_2_-FA-ZnPc) is irradiated with UV or visible light, the dye attached to TiO_2_ is photoexcited, promoting electron transfer from its valence band to its conduction band. Electrons in the dye’s conduction band are injected into the conduction band of TiO_2_, while the oxidized dye is regenerated by oxidizing adsorbed molecules (water and/or O_2_) on the TiO_2_ surface, completing the electronic cycle.

**Scheme 4:**
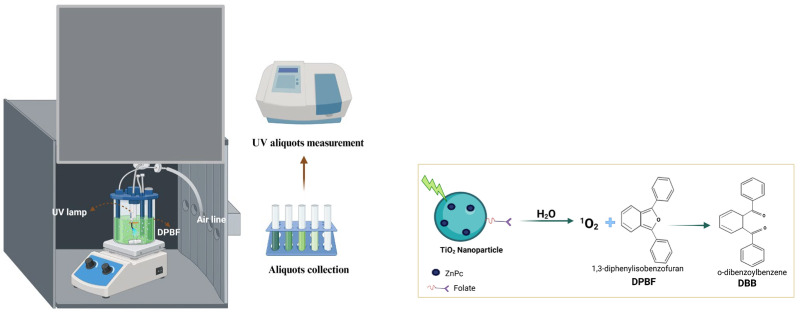
This is a schematic representation of the photocatalytic test using DPBF. This is the proposed reaction mechanism between the produced singlet oxygen and DPBF.

An in situ study was performed using the DPBF probe to evaluate ROS generation by TiO_2_. DPBF (Scheme 4) is a widely used fluorescent probe for ROS detection, undergoing oxidative bleaching to form the colorless compound 1,2-dibenzoylbenzene (DBB) (Scheme 4) [49]. DPBF exhibits a characteristic absorption band near 415 nm, which disappears upon rapid reaction with singlet oxygen (^1^O_2_) (Figure 9). Results indicate that TiO_2_ degrades DPBF faster than TiO_2_-FA and TiO_2_-FA-ZnPc. However, folic acid functionalization is essential for achieving TiO_2_ materials that target specific cells. Meanwhile, ZnPc tends to aggregate in solution and requires stabilization by a high-surface-area support that can be functionalized to disperse ZnPc effectively and facilitate ROS production.

BJ cells, derived from neonatal male foreskin, are human fibroblasts commonly used in biomedical research on aging, cancer, and viral infections. In this study, BJ cells served as a control since they originate from healthy tissue and are free of disease-related alterations such as cancer. As shown in Figure 11, fibroblast viability was not affected after exposure to the various titania materials for up to 72 h.

LN18 and U251 human glioblastoma (GB) cells are widely used in neuro-oncology research. The LN18 cell line was isolated in 1976 from the right temporal lobe of a 65-year-old White male glioblastoma patient [50]. The U-251 MG cell line originated from a 75-year-old Caucasian male. These cell lines were used here to assess the cytotoxicity of the synthesized TiO_2_-based materials. MTT assay demonstrated that all materials exhibited acceptable biocompatibility in fibroblasts at all tested concentrations and in glioma cells at low to moderate concentrations. Specifically, cell viability remained above 80% during the first 24 h of exposure at concentrations up to 100 µg/mL, indicating good tolerance under physiological conditions without irradiation.

**Scheme 5:**
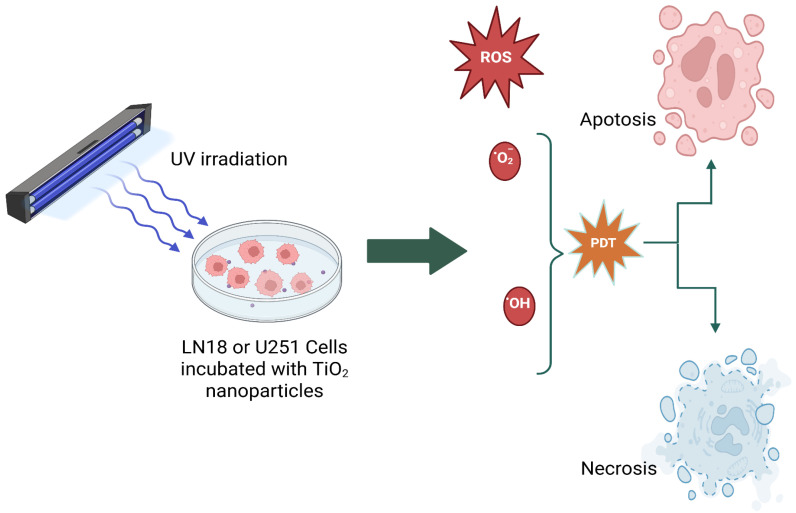
A schematic representation of photodynamic therapy, which is used to kill glioma cells (LN18 or U251 cells).

Scheme 5 schematically illustrates that when cancer cells are treated with different TiO_2_ nanoparticle systems and exposed to UV light, the resulting ROS induce cell death mainly via apoptosis, necrosis, and/or autophagy.

## 5. Conclusions

TiO_2_ nanoparticles were synthesized via the sol–gel method, exhibiting desirable physicochemical properties such as a large surface area, mesoporous structure, and a mixed amorphous–anatase phase. These nanoparticles showed a characteristic TiO_2_ bandgap energy of 3.46 eV, which was retained after functionalization with folic acid. Incorporation of ZnPc into the TiO_2_-FA matrix yielded a multifunctional nanocomposite combining the properties of folic acid and ZnPc.

These materials exhibited enhanced ROS generation, as demonstrated by the rapid degradation of DPBF to the non-colored compound DBB. The nanomaterials were shown to be non-toxic at all concentrations to fibroblast cells. However, they were non-toxic only at low concentrations when LN18 and U251 glioma cells were treated, while higher doses resulted in a reduction in cell viability. Photoactivity assays revealed that cell death depended on both nanomaterial concentration and duration of UV light exposure. Notably, exposure to the nanomaterials alone did not affect LN18 and U251 cells compared to cells exposed to UV light without nanoparticles.

Looking ahead, this experimental research focuses on the in vitro response of TiO_2_-FA-ZnPc under visible light irradiation. Planned biological assays will include both glioma and normal cell lines. In addition to DPBF, DCFH-DA will be used to detect singlet oxygen (^1^O_2_) generation in vitro. Finally, we aim to determine the type of cell death induced by treatments with or without light irradiation.

## Figures and Tables

**Table 1:** Textural and bandgap values of the different TiO_2_, TiO_2_-AF, and TiO_2_-AF-ZnPc materials. *S_BET_* is the surface area, *P_D_* is the pore diameter, and *P_V_* is the pore volume.

Sample	S_BET_ * (m^2^/g)	P_D_ ** (nm)	P_V_ ** (cc/g)	Bandgap (eV)
TiO_2_	602	5.35	0.6564	3.46
TiO_2_-AF	706	4.07	0.7199	2.38
TiO_2_-AF-ZnPc	675	4.90	0.8289	2.38

* Obtained by the adsorption isotherm and using the BETH method, ** obtained from the desorption isotherm and using the BJH method.

**Table 2:** Weight loss % at different temperature intervals of the TiO_2_, TiO_2_-FA, and TiO_2_-FA-ZnPc samples.

Sample	Weight Loss (%)			
	25–150 °C	150–300 °C	300–426 °C	426–600 °C
TiO_2_	14	8		
TiO_2_-AF	14	8	3	
TiO_2_-AF-ZnPc	13	9	3	2

## Data Availability

The original contributions presented in this study are included in the article. Further inquiries can be directed to the corresponding author.

## Abbreviations

The following abbreviations are used in this manuscript:

TiO_2_	Titanium dioxide
ROS	Reactive oxygen species
PDT	Photodynamic therapy
DNA	Deoxyribonucleic acid
UV	Ultraviolet
PS	Photosensitizer
PSES	Photosensitizer excited state
PSET	Photosensitizer excited triplet
WHO	World Health Organization
TMZ	Temozolomide
BBB	Blood–brain barrier
ZnPc	Zinc Phthalocyanine
FA	Folic acid
DPBF	1,3-Diphenylisobenzofuran
SEM	Scanning electron microscopy
HR-TEM	High-resolution transmission electron microscopy
XRD	X-ray diffraction
TGA	Thermogravimetric analysis
VIS	Visible
EDS	Energy-dispersive spectroscopy
MTT	Tetrazolium bromide
FBS	Fetal bovine serum
DMEM	Dulbecco’s Modified Eagle
HOMO	Highest occupied molecular orbital
LUMO	Less unoccupied molecular orbital
Eg	Bandgap energy
BET	Brunauer–Emmett–Teller method
BJH	Barrett–Joyner–Halenda method
